# Fatty acids and nutritional components of the seed oil from Wangmo red ball *Camellia oleifera* grown in the low-heat valley of Guizhou, China

**DOI:** 10.1038/s41598-022-20576-y

**Published:** 2022-10-03

**Authors:** Li Long, Chao Gao, Jie Qiu, Lu Yang, Hongli Wei, Yunchao Zhou

**Affiliations:** Institute for Forest Resources and Environment of Guizhou, Key Laboratory of Forest Cultivation in Plateau Mountain of Guizhou Province, College of Forestry, Guizhou University, Jiaxiu South Road, Guiyang, 550025 China

**Keywords:** Plant breeding, Fruiting

## Abstract

Wangmo red ball *Camellia oleifera* is the main *Camellia* species cultivated for oil in the low-heat valley of Guizhou, China. In this study, we evaluated the comprehensive nutritional value of Wangmo *C. oleifera* seed oil through fatty acid and nutritional component analyses. Twenty excellent Wangmo *C. oleifera* plants with stable yield and disease resistance were selected from the *Camellia oleifera* germplasm resource garden in the low-heat valley site of Guizhou University. The unit crown yield, fatty acid content of the seed oil, fatty acid composition and functional nutrients were determined, and the oil quality was comprehensively evaluated using principal component analysis. The fatty acid content of *C. oleifera* seed oil was 35.03–53.47%, suggesting likely popularization and wide application prospects. The fatty acids included SFAs, MUFAs and PUFAs, and the oleic acid content was 80%, indicating a highly stable and nutritious oil. The oil was also rich in carotenoids, polyphenols, flavonoids, β-sitosterol, squalene and α-Ve, with average content of 7.404 mg/kg, 16.062 mg/kg, 0.401 g/100 g, 265.087 mg/kg, 129.315 mg/kg and 21.505 mg/100 g, respectively. However, the correlations among the nutritional indices were weak. PCA showed that germplasms GH7, GH43, GH28, GH8 and GH31 exhibited the top five nutritional qualities. The rankings in this study provide data for identifying excellent Wangmo *C. oleifera* plants with high nutritional quality. Additionally, this study provides a valuable reference for the research and development of high-end edible oil and a theoretical basis for the development of economic forest species in low-heat valley areas across the world.

## Introduction

*Camellia oleifera* is a small evergreen tree belonging to the family Theaceae *Camellia*, which is a unique high-quality woody plant in China^1^. The oil extracted from *C. oleifera* seed kernels has high nutritional value, and its fatty acid composition is very similar to that of olive oil. As a result, it has been dubbed "oriental olive oil"^2^. In China, approximately one-seventh of the population uses tea seed oil for cooking. Tea seed oil contains more than 80% unsaturated fatty acids, such as oleic acid, linoleic acid and linolenic acid. It is characterized by its unique flavor, long storage life and easy absorption by the human body^3,4^. Tea seed oil is also rich in functional active substances, such as carotenoids, polyphenols, flavonoids, β-sitosterol, squalene and vitamins^5^, and these substances have a variety of physiological functions, such as delaying aging and inhibiting free radicals^6,7^. Long-term consumption of tea seed oil can effectively prevent coronary heart disease^7^, arteriosclerosis^8^, skin diseases^9^ and tumors^10^.

The low-heat valley area of Guizhou Province is located in the sloped transition from the Yunnan-Guizhou Plateau to the low mountains and hills in Guangxi, China. The area has a warm and humid subtropical monsoon climate. Wangmo red ball *Camellia oleifera* is a special type of *Camellia oleifera* distributed in this area. It has been rated as an excellent farm variety in China since the 1970s and 1980s^11^. Wangmo *C. oleifera* is characterized by large fruits, smooth and red peels, strong disease and insect resistance, early fruiting and high yield; moreover, it possesses excellent adaptability to the mountain climate of dry and hot river valleys. In recent years, researchers have examined a variety of aspects of various types of *C. oleifera* seed oil, including its nutritional components^9,12^, functions^7^ and applications^13^. The influence of the oil-making process on the quality of seed oil has also been investigated^14^. As the value of Wangmo *C. oleifera* has gradually become clear, the number research reports on its characteristics has also increased. However, only the analysis of fruit characteristics has been reported in the literature^15^, and the reports on oil quality have not been systematic and comprehensive. Thus, in this study, research regarding the quality of the oil of Wangmo *C. oleifera* was carried out to provide basic data for enhancing the quality of the oil.

In recent years, with the increasing demand for fast food and the high fat content diet predominantly found in modern countries, the importance of finding more high-end edible oils from natural resources has been highlighted. An in-depth understanding of the oil quality of Wangmo *C. oleifera* would form the basis for selective breeding of this species with the goal of generating of high-quality edible oil. In addition, a comprehensive assessment of Wangmo *C. oleifera* plants would serve as the basis for the utilization of excellent germplasm resources. A number of methods have been used for the comprehensive assessment of excellent plants, such as the fuzzy comprehensive evaluation method^16^, the integrated index method^17^, principal component analysis (PCA)^18^, the reasonableness and satisfaction index method^19^ and the gray correlation degree method^20^. All these methods have their own merits and drawbacks. The quality of oil fat is measured by several indices, and therefore, univariable methods fail to establish models for the relationship among samples. For the gray correlation degree method and the fuzzy comprehensive evaluation method, determination of the weight coefficients of the factors plays a critical role in assessing the quality of *Camellia* seed oils. Thus, to provide a reliable theoretical basis, researchers must determine the weight coefficient distribution of the factors from multiple perspectives. PCA is a statistical analysis method that reduces the dimension of multiple groups of data to several independent information groups. The advantage of this method over other evaluation methods is that it can eliminate the interference among the evaluation indices without the need to artificially assign weights or create ideal weights for the factors to ensure the objectivity of the evaluation. Similar to the reasonableness and satisfaction index method, PCA also adopts weighted summation to calculate the comprehensive score; however, the weights originate from the ratio of the contribution rate of the extracted principal component to the total contribution rate. For this reason, it has been extensively employed in the comprehensive evaluation of drugs^21^, grain^22^, water^23^ and *Camellia*.

In this study, we performed a comprehensive assessment of the fatty acid composition and functional nutrients of Wangmo *C. oleifera* seed oil using PCA. The plants were 20 disease/pest-resistant Wangmo *C. oleifera* germplasm resources of high and stable production based on five-year observations. The final results of this study identified germplasms with excellent comprehensive performance as candidate resources for cultivar selection and also provide basic data for the development of high-end edible oils, establishing a theoretical basis for the development of economic forest species in low-heat valley areas across the world.

## Results

### Yield per unit crown width

As shown in Table 1 (supplementary File 1), the yields per unit crown width of the 20 studied plants ranged from 4.46 to 7.72 kg/m^2^, with an average of 5.57 kg/m^2^. The yield per unit crown width of GH28 was the highest (7.72 kg/m^2^), while that of GH61 was the lowest (4.46 kg/m^2^).

**Table 1:** Crown width yield and ranking of 20 superior strains of Wangmo red ball *Camellia oleifera.*

Plant	Total weight of fresh fruit (kg)	Crown width (m^2^)	Yield per unit crown width (kg/m^2^)	Ranking
GH28	27.8	2.0 × 1.8	7.72	1
GH30	22.2	1.8 × 1.8	6.85	2
GH36	17.9	1.7 × 1.6	6.58	3
GH43	23.5	1.9 × 1.9	6.51	4
GH7	23.2	2.0 × 1.9	6.11	5
GH40	19.5	1.8 × 1.8	6.02	6
GH58	17.8	1.8 × 1.7	5.82	7
GH57	19.8	1.9 × 1.8	5.79	8
GH52	24.2	2.1 × 2.0	5.76	9
GH62	22.1	2.0 × 2.0	5.53	10
GH8	18.9	2.0 × 1.8	5.25	11
GH34	19.8	2.0 × 1.9	5.21	12
GH32	16.5	1.7 × 1.9	5.11	13
GH53	16.5	1.8 × 1.8	5.09	14
GH31	19.7	2.0 × 2.0	4.93	15
GH33	14.9	1.7 × 1.8	4.87	16
GH21	20.1	2.1 × 2.0	4.79	17
GH59	16.5	1.9 × 1.9	4.57	18
GH56	14.5	1.8 × 1.8	4.48	19
GH61	17.8	2.1 × 1.9	4.46	20
Average	19.4	1.9 × 1.8	5.57	–

### Fat content

**Figure 1:**
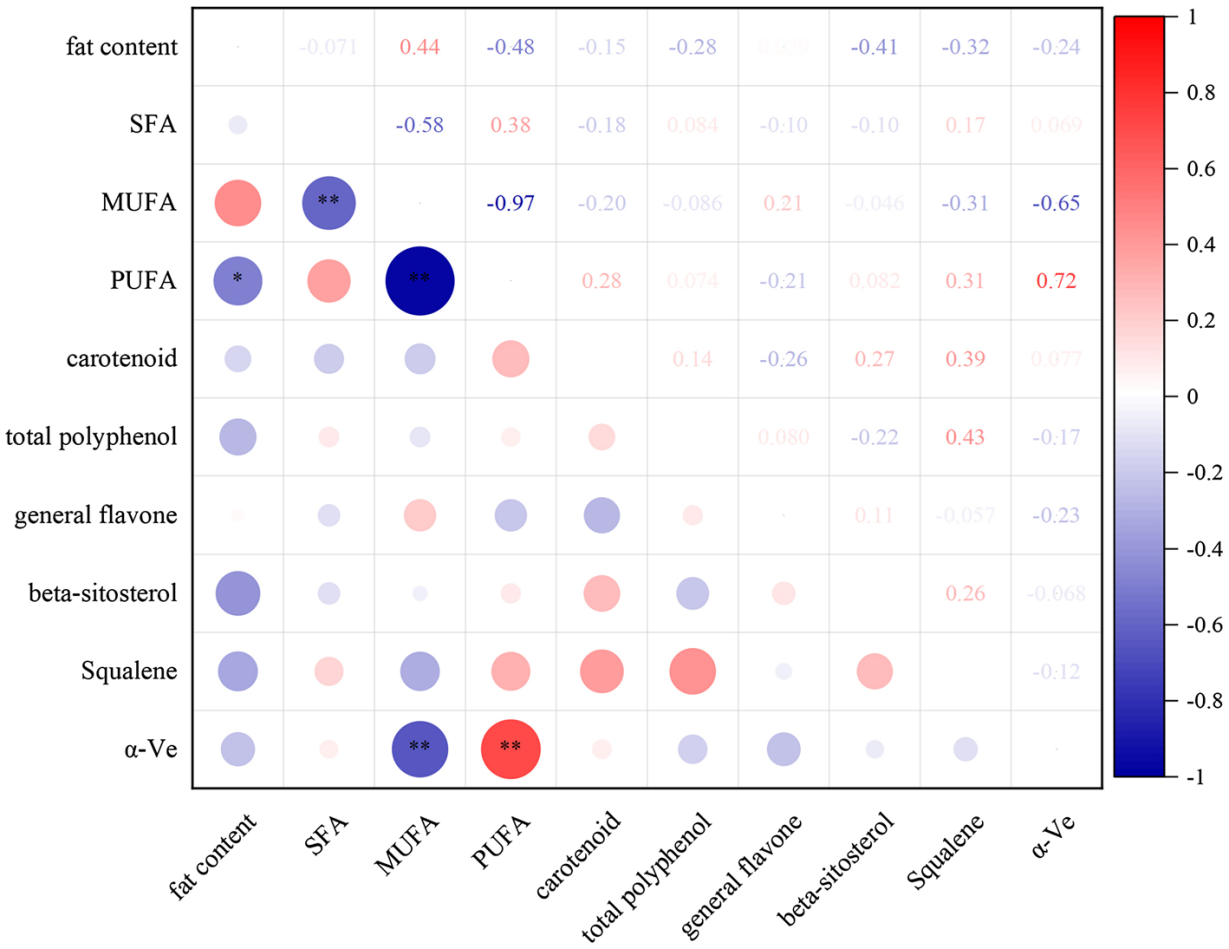
Seed fat contents of 20 strains of Wangmo red ball *Camellia oleifera. Note* the same letters in the same line indicate no significant difference (*P* > 0.05), while different lowercase letters in the same line indicate a significant difference (*P* < 0.05).

The fat contents of the 20 plants differed greatly (Fig. 1, Supplementary File 1), ranging from 35.03 to 53.47%, with an average of 47.03%. The highest fat content was observed in GH33 (53.47%), and the lowest was found in GH28 (35.03%). Only one plant had a fat content lower than 40%, 15 plants had fat contents between 40 and 50%, and 4 plants had fat contents between 50 and 60%.

### Fatty acid composition

As shown in Table 2 (supplementary File 1), the fatty acids of Wangmo *C. oleifera* included palmitic acid, palmitoleic acid, stearic acid, oleic acid, linoleic acid, linolenic acid and cis-11-eicosenoic acid, and the plants differed significantly in fatty acid composition (*P* < 0.05). Both saturated fatty acids (SFAs) and unsaturated fatty acids (UFAs) were found in *C. oleifera* seed oil; the UFAs included both monounsaturated fatty acids (MUFAs) and polyunsaturated fatty acids (PUFAs), with MUFAs as the main type.

**Table 2:** Fatty acid components in the seed oil of 20 Wangmo red ball *Camellia oleifera* germplasms.

Plant	Palmitic acid C16:0	Palmitoleic acid C16:1	Stearic acid C18:0	Oleic acid C18:1	Linoleic acid C18:2	Linolenic acid C18:3	Cis-11-eicosenoic acid C20:1	SFA	MUFA	PUFA
GH7	8.290 ± 0.040f	0.087 ± 0.001f	1.360 ± 0.010p	79.833 ± 0.058 k	9.500 ± 0.020f	0.355 ± 0.045e	0.575 ± 0.004 g	9.650	80.495	9.855
GH8	9.040 ± 0.040a	0.045 ± 0.003j	1.760 ± 0.020 l	77.967 ± 0.058q	10.233 ± 0.058c	0.351 ± 0.009e	0.604 ± 0.019 cd	10.800	78.616	10.584
GH21	8.610 ± 0.010c	0.044 ± 0.001j	1.900 ± 0.010 k	78.700 ± 0.000n	9.783 ± 0.006e	0.351 ± 0.004e	0.612 ± 0.001bc	10.510	79.356	10.134
GH28	7.960 ± 0.010 h	0.087 ± 0.001f	2.240 ± 0.010 g	79.167 ± 0.058 m	9.440 ± 0.010f	0.507 ± 0.049a	0.599 ± 0.002de	10.200	79.853	9.947
GH30	7.417 ± 0.006 l	0.073 ± 0.001 g	2.557 ± 0.006a	81.400 ± 0.000 h	7.667 ± 0.006 g	0.353 ± 0.001e	0.535 ± 0.002jk	9.974	82.008	8.020
GH31	7.830 ± 0.030i	0.049 ± 0.005j	1.570 ± 0.010n	80.067 ± 0.058j	9.463 ± 0.058f	0.477 ± 0.038b	0.544 ± 0.002i	9.400	80.660	9.940
GH32	7.290 ± 0.010 m	0.057 ± 0.003i	2.480 ± 0.020b	82.600 ± 0.000e	6.767 ± 0.006i	0.292 ± 0.002fj	0.514 ± 0.002 mn	9.770	83.171	7.059
GH33	8.760 ± 0.010b	0.092 ± 0.002f	2.267 ± 0.006f	80.600 ± 0.000i	7.523 ± 0.006 h	0.270 ± 0.005j	0.488 ± 0.004o	11.027	81.180	7.793
GH34	6.780 ± 0.010p	0.099 ± 0.001e	2.100 ± 0.000i	83.433 ± 0.058b	6.657 ± 0.049j	0.344 ± 0.002e	0.587 ± 0.001f	8.880	84.119	7.001
GH36	8.560 ± 0.010d	0.006 ± 0.000 m	1.460 ± 0.000o	78.200 ± 0.100p	10.900 ± 0.100a	0.310 ± 0.010f	0.564 ± 0.000 h	10.020	78.770	11.210
GH40	7.653 ± 0.006 k	0.058 ± 0.004i	2.133 ± 0.012 h	79.400 ± 0.000 l	9.977 ± 0.021d	0.291 ± 0.001fj	0.488 ± 0.002o	9.786	79.946	10.268
GH43	7.323 ± 0.029 m	0.107 ± 0.001d	2.217 ± 0.032 g	83.033 ± 0.058d	6.443 ± 0.006 k	0.349 ± 0.008e	0.528 ± 0.002kl	9.540	83.668	6.792
GH52	8.500 ± 0.000e	0.121 ± 0.001c	1.750 ± 0.010 l	78.533 ± 0.058o	10.167 ± 0.058c	0.403 ± 0.004 cd	0.526 ± 0.005 lm	10.250	79.180	10.570
GH53	8.050 ± 0.010 g	0.065 ± 0.000hi	1.667 ± 0.012 m	78.433 ± 0.153o	10.767 ± 0.153b	0.425 ± 0.003c	0.593 ± 0.003ef	9.717	79.091	11.192
GH56	7.777 ± 0.015j	0.061 ± 0.001hi	2.383 ± 0.029d	83.533 ± 0.058b	5.417 ± 0.015 m	0.288 ± 0.002fj	0.541 ± 0.002ij	10.160	84.135	5.705
GH57	7.037 ± 0.006o	0.037 ± 0.002 k	2.320 ± 0.010e	83.300 ± 0.000c	6.347 ± 0.012 l	0.343 ± 0.002e	0.617 ± 0.002b	9.357	83.954	6.690
GH58	7.967 ± 0.015 h	0.024 ± 0.001 l	2.443 ± 0.006c	79.200 ± 0.000 m	9.420 ± 0.010f	0.302 ± 0.001fj	0.644 ± 0.001a	10.410	79.868	9.722
GH59	7.213 ± 0.021n	0.460 ± 0.010b	2.230 ± 0.010 g	82.400 ± 0.100f	6.800 ± 0.100i	0.390 ± 0.005d	0.507 ± 0.002n	9.443	83.367	7.190
GH61	6.773 ± 0.021p	0.640 ± 0.010a	2.560 ± 0.010a	83.733 ± 0.058a	5.503 ± 0.025 m	0.295 ± 0.001fj	0.495 ± 0.001o	9.333	84.868	5.798
GH62	8.063 ± 0.064 g	0.106 ± 0.001d	1.923 ± 0.006j	81.633 ± 0.058 g	7.480 ± 0.010 h	0.273 ± 0.000j	0.521 ± 0.001 lm	9.986	82.260	7.753
Average	7.845	0.116	2.066	80.758	8.313	0.348	0.554	9.911	81.428	8.661
CV/%	8.19	129.64	17.46	2.47	21.32	18.24	8.30	5.24	2.52	20.75

The data in the table are the mean ± standard deviation; the same letters in the same line indicate no significant difference (*P* > 0.05), while different lowercase letters in the same line indicate a significant difference (*P* < 0.05). CV: coefficient of variation.

The SFAs included palmitic acid and stearic acid and the SFA content of the plants ranged from 8.880 to 11.027%. The content of palmitic acid ranged from 6.773% (GH61) to 9.040% (GH8), with an average of 7.845%. The content of stearic acid ranged from 1.360% (GH7) to 2.560% (GH61), with an average of 2.066%. The MUFAs included oleic acid, palmitoleic acid and cis-11-eicosenoic acid, and the MUFU content of the plants ranged from 78.616 to 84.868%. Oleic acid was the main type of MUFA; the content of oleic acid ranged from 77.967% (GH8) to 83.733% (GH61), with an average of 80.785%. The highest palmitoleic acid content was observed in GH61 (0.640%), while the lowest was observed in GH36 (0.006%). The content of cis-11-eicosenoic acid ranged from 0.488 to 0.644%. The PUFA content of the selected plants ranged from 5.705 to 11.210% and included linoleic acid and linolenic acid. The content of linoleic acid ranged from 5.417% (GH56) to 10.900% (GH36), with an average of 8.313%. The content of linolenic acid ranged from 0.270% (GH33) to 0.507% (GH28), and the average was 0.348%.

The plants of Wangmo *C. oleifera* also differed greatly in the coefficients of variation of (CV) the fatty acid components, and the CVs ranked from highest to lowest were as follows: C16:1 (129.64%), C18:2 (21.32%), C18:3 (18.24%), C18:0 (17.46%), C20:1 (8.30%), C16:0 (8.19%), and C18:1 (2.47%). The CVs of the SFA, MUFA and PUFA contents were 5.24%, 2.52% and 20.75%, respectively. The SFA content in all samples was below 12%, while the UFA content was as high as 90%, indicating that Wangmo *C. oleifera* seed oil is an excellent edible oil rich in UFAs.

### Nutrient composition analysis

Carotenoids, total polyphenols, total flavonoids, β-sitosterol, squalene and vitamin E were the main nutrients in Wangmo *C. oleifera* in the low-heat valley (Table 3, Supplementary File 1), and their contents differed significantly (*P* < 0.05). Among the 20 germplasms considered, the carotenoid content ranged from 5.067 mg/kg (GH34) to 11.850 mg/kg (GH43), with an average of 7.404 mg/kg. Polyphenols are natural antioxidants that scavenge free radicals in the body and have strong anti-inflammatory and antibacterial effects^24,25^. The content of total polyphenols varied from 10.333 mg/kg (GH33) to 23.800 mg/kg (GH58), with an average of 16.062 mg/kg. GH7 had the lowest total flavonoid content (0.180 g/100 g), while GH59 had the highest (0.630 g/100 g). The average total flavonoid content was 0.401 g/100 g. GH58 had a significantly higher squalene content than any other germplasm (230.567 mg/kg), whereas GH62 had the lowest squalene content (61.800 mg/kg). The average squalene content of all germplasms was 129.315 mg/kg. Vitamin E has four configurations: α-Ve, β-Ve, γ-Ve and δ-Ve. However, only α-Ve was detected in Wangmo *C. oleifera*. The most bioactive structure in vitamin E^26^, α-Ve can promote fertility, improve lipid metabolism, promote the secretion of sex hormones, and delay aging^27^. In this study, GH36 had the highest α-Ve content (28.767 mg/100 g), while GH43 had the lowest content (16.457 mg/100 g). The average α-Ve content of all germplasms was 21.505 mg/100 g. Therefore, Wangmo *C. oleifera* seed oil can be regarded as a natural α-Ve supplement. As shown in Table 3, the CVs of the six nutrients in the 20 germplasms ranged from 16.79 to 33.86%. β-Sitosterol had the smallest CV (16.79%), indicating that β-sitosterol had the smallest improvement potential. In contrast, squalene had the highest CV (33.86%), which indicated a rather high improvement potential. According to the CV values, the nutritional substances were rated in terms of improvement potential as follows: squalene > total flavonoids > total polyphenols > carotenoids > α-Ve > β-sitosterol.

**Table 3:** Nutritional composition of seeds and oils of 20 strains of Wangmo red ball *Camellia oleifera.*

Plant	Carotenoids (mg/kg)	Total polyphenols (mg/kg)	Total flavonoids (g/100 g)	β-sitosterol (mg/kg)	Squalene (mg/kg)	α-Ve (mg/100 g)
GH7	9.617 ± 0.040b	15.497 ± 0.021 k	0.180 ± 0.00j	277.400 ± 1.039 g	155.200 ± 1.054e	21.633 ± 0.153e
GH8	7.020 ± 0.020 h	16.110 ± 0.101j	0.273 ± 0.015 h	283.500 ± 0.300f	202.633 ± 1.026b	21.300 ± 0.100ef
GH21	6.030 ± 0.044 m	18.060 ± 0.165 g	0.227 ± 0.015i	181.533 ± 0.929q	74.733 ± 0.981p	27.467 ± 0.416bc
GH28	6.950 ± 0.010 h	18.913 ± 0.015e	0.610 ± 0.010ab	378.233 ± 0.586a	167.333 ± 0.551d	20.700 ± 0.100 fg
GH30	6.127 ± 0.040klm	21.440 ± 0.010b	0.427 ± 0.012e	231.733 ± 0.666n	78.667 ± 0.208o	21.133 ± 0.115ef
GH31	8.957 ± 0.031d	10.600 ± 0.021q	0.433 ± 0.015e	285.367 ± 0.321f	149.300 ± 0.624 g	27.733 ± 0.208b
GH32	6.650 ± 0.061i	12.297 ± 0.0503o	0.433 ± 0.012e	251.800 ± 0.529 k	102.467 ± 0.874 m	18.800 ± 0.173j
GH33	6.310 ± 0.010j	10.333 ± 0.0153r	0.503 ± 0.015 cd	272.233 ± 0.208 h	110.500 ± 0.400 l	19.503 ± 0.015i
GH34	5.067 ± 0.021n	14.367 ± 0.208 m	0.483 ± 0.035d	291.533 ± 0.862e	110.233 ± 0.379 l	20.267 ± 0.252gh
GH36	8.230 ± 0.030e	17.490 ± 0.010 h	0.383 ± 0.006f	256.400 ± 0.361j	96.500 ± 0.436n	28.767 ± 0.153a
GH40	7.577 ± 0.055f	13.750 ± 0.030n	0.320 ± 0.010 g	328.500 ± 0.361b	141.567 ± 0.493i	26.900 ± 1.153c
GH43	11.850 ± 0.176a	20.790 ± 0.127c	0.237 ± 0.006i	309.600 ± 1.015c	176.433 ± 0.404c	16.457 ± 1.010 l
GH52	8.227 ± 0.031e	18.720 ± 0.0608f	0.513 ± 0.031 cd	259.900 ± 4.440i	150.600 ± 0.265f	17.700 ± 0.200 k
GH53	9.070 ± 0.072c	12.257 ± 0.0723o	0.363 ± 0.015f	271.500 ± 0.100 h	112.800 ± 0.100 k	25.667 ± 0.321d
GH56	6.207 ± 0.025 k	14.633 ± 0.153 l	0.373 ± 0.015f	298.033 ± 0.503d	112.367 ± 0.473 k	17.633 ± 0.153 k
GH57	6.177 ± 0.023kl	17.200 ± 0.044i	0.300 ± 0.010gh	234.133 ± 1.747 m	147.433 ± 0.404 h	18.233 ± 0.058jk
GH58	7.140 ± 0.101 g	23.800 ± 0.200a	0.450 ± 0.010e	190.533 ± 0.907p	230.567 ± 0.862a	21.633 ± 0.306e
GH59	7.603 ± 0.021f	19.523 ± 0.0208d	0.630 ± 0.010a	230.433 ± 0.058n	137.500 ± 0.608j	19.767 ± 0.153hi
GH61	7.180 ± 0.030 g	14.640 ± 0.0346 l	0.600 ± 0.020b	241.200 ± 0.608 l	67.667 ± 1.531q	18.500 ± 0.100j
GH62	6.090 ± 0.026 lm	10.830 ± 0.085p	0.280 ± 0.010 h	228.167 ± 1.595o	61.800 ± 0.400r	20.300 ± 0.300gh
Average	7.404	16.062	0.401	265.087	129.315	21.505
CV/%	20.74	23.17	31.89	16.79	33.86	16.97

The data in the table are the mean ± standard deviation; the same letters in the same line indicate no significant difference (*P* > 0.05), while different lowercase letters in the same line indicate a significant difference (*P* < 0.05). CV stands for coefficient of variation.

### Correlation analysis of nutritional indices

**Figure 2:**
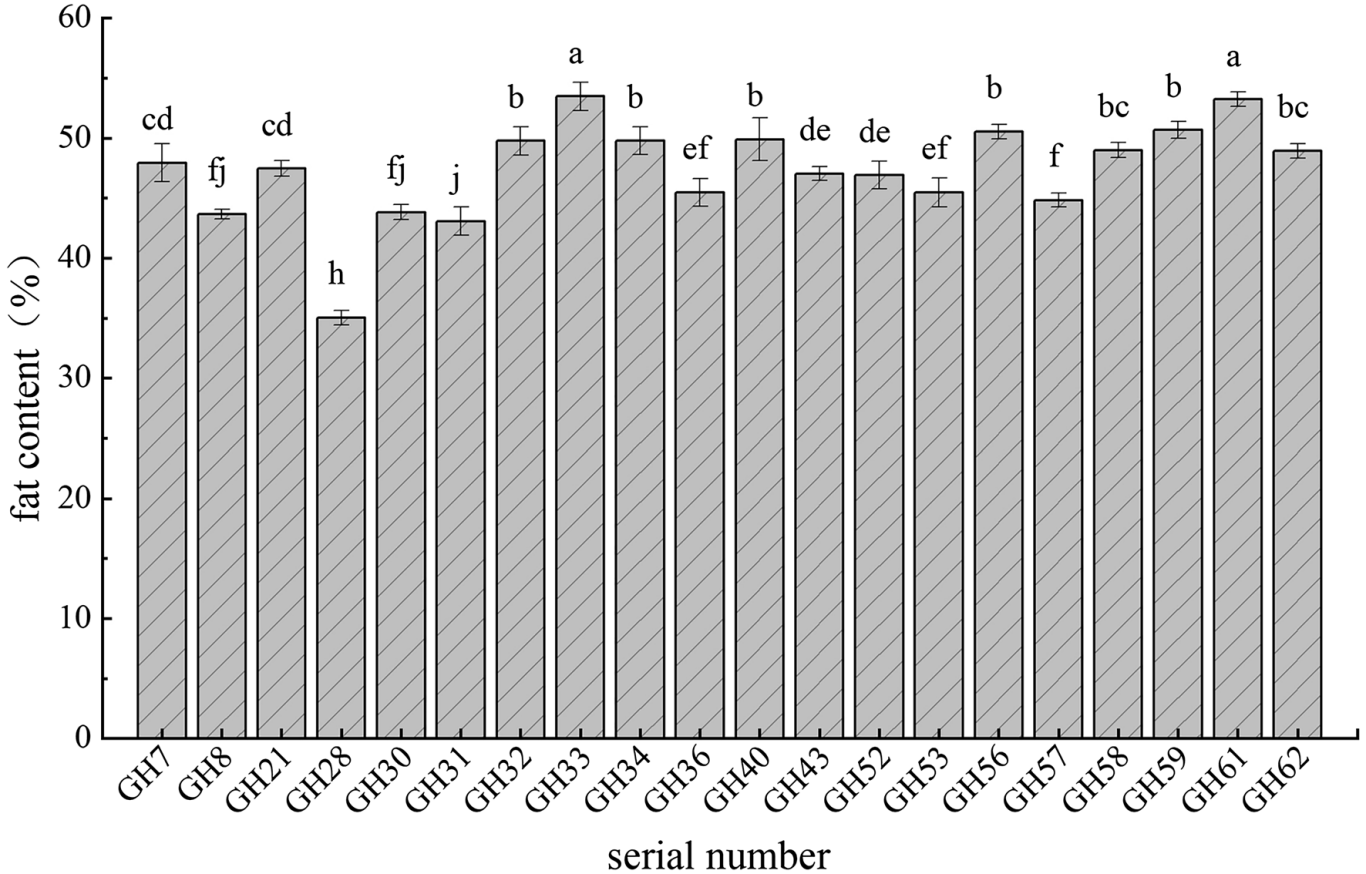
Correlation matrix of quality indices of Wangmo red ball *Camellia oleifera*. *Note* *: significant correlation was found at the level of 0.05 (bilateral); **: extremely significant correlation was found at the 0.01 level (bilateral).

The correlation analysis of the 11 quality indices of Wangmo *C. oleifera* seed oil in the low-heat valley of Guizhou Province showed variability in the correlations among them (Fig. 2). There was a significant negative correlation between fat and PUFA contents, and the correlation coefficient was − 0.48. The SFA content exhibited a very significant negative correlation with the MUFA content, with a correlation coefficient of − 0.58. The MUFA content had a very significant negative correlation with the PUFA content and α-Ve, with correlation coefficients of − 0.97 and − 0.64, respectively.

The positive correlation between PUFA content and α-Ve was significant, with a correlation coefficient of 0.71. Although other nutritional indices also had different degrees of correlation, none of the correlations were statistically significant. The nutritional components of different Wangmo *C. oleifera* germplasms varied, and the correlations between the components also differed. Therefore, evaluating the nutritional quality of Wangmo *C. oleifera* seed oil only based on a single index would be an incomplete assessment.

### PCA

In the evaluation of the nutritional components of Wangmo red ball *Camellia oleifera* seed oil in the low-heat valley of Guizhou, a comprehensive, systematic and scientific assessment of the quality of *Camellia oleifera* seed oil should be considered, focusing on more than one or even several nutritional components. PCA is an effective method for comprehensive evaluation. It is a multivariate statistical analysis method that can select a small number of important variables via the linear transformation of multiple variables^28^. In this study, the fat, SFA, MUFA, PUFA, carotenoid, total polyphenol, total flavonoid, β-sitosterol, squalene and α-Ve contents were selected for PCA. Among these considered indices, the lower the value of saturated fatty acids was, the better the quality of the seed oil. To ensure that all parameters were positively correlated with the quality of seed oil, the saturated fatty acid was set to a negative number, and the original values of each index were standardized.

Four principal components with eigenvalues greater than 1 were extracted by PCA. As shown in Table 4, the cumulative variance contribution rate of the first four principal components was 76.734%, indicating that these components contained most of the information for the nutritional indices of Wangmo red ball *C. oleifera* seed oil. These four components could be used to represent the original 10 evaluation indices for the analysis and evaluation of the nutritional components of Wangmo red ball *C. oleifera* seed oil.

**Table 4:** Component load matrix of each nutritional index.

Index	PC1	PC2	PC3	PC4
Fat	− 0.609	− 0.368	− 0.100	0.348
SFA	− 0.451	0.270	0.550	0.196
MUFA	− 0.947	0.228	0.105	0.087
PUFA	0.949	− 0.183	0.039	− 0.043
Carotenoids	0.366	0.481	0.368	0.522
Total polyphenols	0.190	0.502	− 0.657	0.189
Total flavonoids	− 0.302	0.194	− 0.180	− 0.704
β-sitosterol	0.187	0.510	0.582	− 0.439
Squalene	0.468	0.669	− 0.235	0.110
α-Ve	0.637	− 0.525	0.293	0.021
Characteristic value	3.294	1.799	1.404	1.177
Contribution rate	32.943	17.986	14.036	11.769
Cumulative contribution rate (%)	32.943	50.929	64.965	76.734

As shown in Table 4, PC1 contained the comprehensive indices of fat, SFA, PUFA and α-Ve contents. The eigenvalue of PC1 was 3.294, and its contribution rate was 32.943%. Among the indices, PUFAs contributed the most to PC1, with a load value of 0.949. The characteristic value of PC2 was 1.799, with a contribution rate of 17.986%. This component only contained squalene, with a load value of 0.669. PC3 included SFAs, total polyphenols and β-sitosterol, with a characteristic value of 1.404. The load values of the three factors were 0.550, − 0.657 and 0.582, respectively. PC4 had a high negative correlation with total flavonoids, and the load value was − 0.704, indicating that total flavonoids was the main evaluation index in the fourth component.

With the proportion of the contribution rate corresponding to the four principal components in the total contribution rate as the weight, the comprehensive evaluation function of *C. oleifera* seed oil in the low-heat valley area was as follows: F = 0.429PC1 + 0.234PC2 + 0.183PC3 + 0.153PC4. The principal component score, comprehensive score and ranking of each excellent germplasm were calculated (Table 5). In PC1, GH8 scored the highest (2.510). GH43 scored the highest in PC2 and PC4 (3.194 and 2.299, respectively). The highest score in PC3 was that of GH31 (2.126). The top five nutritional qualities among the 20 excellent single plant tea seed oils were observed for GH7, GH43, GH28, GH8 and GH31, among which GH7 and GH43 had the top two comprehensive nutritional values.

**Table 5:** Principal component score, comprehensive score and ranking of all superior strains of Wangmo red ball *Camellia oleifera.*

Plant	Principal component score				Comprehensive score	Rating
	PC1	PC2	PC3	PC4		
GH7	1.134	0.474	0.998	1.769	1.051	1
GH43	− 0.432	3.194	0.707	2.299	1.043	2
GH28	2.036	2.337	0.203	− 3.098	0.983	3
GH8	2.510	0.300	− 0.858	− 0.211	0.958	4
GH31	1.410	− 0.091	2.126	− 0.247	0.935	5
GH53	1.822	− 0.783	1.495	0.231	0.907	6
GH36	2.222	− 1.212	0.383	0.112	0.757	7
GH40	1.335	− 0.540	1.470	− 0.007	0.714	8
GH52	1.079	0.738	− 1.102	− 0.380	0.376	9
GH58	1.325	0.737	− 2.990	0.800	0.316	10
GH21	1.355	− 2.749	− 1.210	0.880	− 0.149	11
GH57	− 1.514	0.729	− 0.316	0.610	− 0.444	12
GH30	− 0.465	− 0.236	− 1.195	− 0.348	− 0.527	13
GH59	− 1.753	0.840	− 0.858	− 0.074	− 0.724	14
GH32	− 1.831	− 0.482	0.275	− 0.178	− 0.875	15
GH62	− 1.303	− 1.879	0.383	0.279	− 0.886	16
GH56	− 2.198	0.177	− 0.045	− 0.311	− 0.957	17
GH34	− 2.436	0.097	0.908	− 0.803	− 0.979	18
GH33	− 0.870	− 1.424	− 0.598	− 1.109	− 0.986	19
GH61	− 3.425	− 0.225	0.225	− 0.215	− 1.514	20

## Discussion

Wangmo red ball *C. oleifera* is a high-quality woody edible oil tree species, but its development and utilization remains in the early stages. The quantitative analysis and evaluation of the seed oil quality indices can provide basic data for the selection of high-quality edible oil. The yield per unit crown width and fat content are important indicators for measuring the yield of *C. oleifera*^29^. The crown width yield of Wangmo red ball *C. oleifera* in the low heat valley of Guizhou was significantly higher than that of "Changlin" series *C. oleifera* (0.76 ~ 0.9 kg/m^2^)^30^ (Table 1). The high performance of Wangmo *C. oleifera* can be attributed to its varieties and is also closely related to the low-heat valley area. This area is located in the hot subtropical climate zone of the low-middle mountain canyon, with alternating dry and wet seasons, and its climatic conditions are conducive to the growth of *C. oleifera* fruit. This study showed that the crown yield of Wangmo *C. oleifera* was negatively correlated with the fat content, which was consistent with the results of Liu Wei^31^ who reported the correlation between yield and kernel fat content. The fat content of *C. oleifera* seed oil directly affects the promotion, utilization and market value of the species. The fat content of olive oil is 43.7% to 56.7%^32^, that of soybean oil is 14.51% to15.51%^33^, that of sunflower oil is 35.1% to 43.0%^34^, and that of mustard seed oil is 33.0 to 38.0%^35^. In this study, the fat content of Wangmo *C. oleifera* seed oil was between 35.03 and 53.47% (Fig. 1). Thus, compared with the fat content of many edible oils, the fat content of Wangmo *C. oleifera* seed oil was high. This finding indicates that Wangmo *C. oleifera* has satisfactory practical value and development potential.

Edible oils rich in MUFAs are easily absorbed by the human body, and the digestibility can exceed 95% (Table 2). These oils can effectively promote human metabolism and reduce the incidence of cardiovascular and cerebrovascular diseases. The composition and content of fatty acids in edible oil can regulate human health. In the fatty acids of Wangmo *C. oleifera*, the main component was UFAs, with a content of approximately 90%; the SFA content was approximately 10%. The composition and content of the fatty acids in Wangmo *C. oleifera* satisfied the requirements for SFA content based on international nutritional standards^36^. The SFA content in tea oil increases with increasing latitude of origin, while the UFA content decreases with increasing latitude^37,38^. In this study, Wangmo *C. oleifera* was obtained from a low-latitude area, and the UFA content was higher than that in plants growing in high-altitude areas^39,40^. Therefore, it is reasonable to assume that the high content of UFAs in Wangmo *C. oleifera* is associated with the low-latitude region. Oleic acid is a single UFA. The content of oleic acid is an important indicator to evaluate the quality of edible oil and to judge the stability of the oil. The high content of oleic acid in tea seed oil means that it has high stability and digestibility, as well as health-promoting effects such as reducing blood pressure, cholesterol and triglycerides. In this study, we found that the content of oleic acid in Wangmo *C. oleifera* oil was 80.79%, which had not been previously reported in the literature. Ceheng *C. oleifera* and Wangmo *C. oleifera* belong to the same *Camellia* species, and they grow in regions with consistent climatic and geographical conditions. According to the literature^39^, Ceheng *C. oleifera* oil has an oleic acid content of 80.19%. Our findings indicate that Wangmo *C. oleifera* produces oil with high stability and high nutritional value. The oleic acid contents in different editable oils vary: 74.20% in olive oil, 58.19% in rapeseed oil, 25.90% in corn oil, 79.71% in sunflower oil, 39.46% in peanut oil, 22.40% in bean oil and 28.06% in pumpkin oil. This study showed that the oleic acid content of Wangmo *C. oleifera* seed soil was as high as 80%, which was higher than that of other edible oils, including the oils produced from different *C. oleifera* species^39–41^. In terms of the oleic acid content, Wangmo *C. oleifera* seed oil has an absolute advantage over ordinary vegetable oil and a higher nutritional value than most *C. oleifera* seed oils. Therefore, based on the analysis of the fatty acid composition and oleic acid content, Wangmo red ball *C. oleifera* seed oil is a high-quality woody edible oil.

Functional components, such as carotenoids, polyphenols, flavonoids, β-sitosterol, squalene and vitamins, can also serve as important indicators for the evaluation of the quality and function of tea oil. According to the current study, the contents of β-sitosterol, α-Ve and total flavonoids in Wangmo red ball *C. oleifera* seed oil were 265.087 mg/kg, 21.505 mg/100 g and 0.401 g/100 g, respectively (Table 3). Among *C. oleifera* varieties, the levels of β-sitosterol, α-Ve and flavonoids in Wangmo *C. oleifera* seed oil were higher than average based on the values reported in the literature^39,40,42^. The content of total flavonoids in Wangmo *C. oleifera* was as high as 0.401 g/100 g in this study, which was far higher than that in *Camellia* (0.360–0.540 mg/g), as reported in the literature^43^. The reason might be related to the growing conditions. Wangmo *C. oleifera* grows in Guizhou, where differences in region of production and species have a great influence on the content of active substances in *Camellia* seed oil. The content of total flavonoids in *Cyclocarya paliurus* exhibits an increase with altitude to a point, followed by a decrease with altitude^44^. In Yang et al.’s study^43^, the *Camellia* material was from Taiwan, and the plants were primarily distributed at an altitude of approximately 2000 m. In contrast, the material in our study was from the Wangmo region in Guizhou, with an altitude of approximately 800 m. *Camellia weiningensis* grows at 1800 m, and its total flavonoid content was 0.540 mg/g^39^. From the perspective of the influence of altitude, our findings regarding Wangmo *C. oleifera* and those reported for other *Camellia* species support the trend in the content of total flavonoids which increases and then decrease with altitude. The climate in Wangmo is humid and hot with sufficient sunlight. studies on the mechanism underlying the formation of functional active substances in oil fat have been limited. Therefore, whether the high content of total flavonoids in Wangmo *C. oleifera* seed oil is related to the unique climate characteristics requires further research. In Wangmo *C. oleifera*, the coefficients of variation of the six nutritional substances studied ranged from 16.79 to 33.86% (Table 2). All of the indices exhibited some improvement potential, and Wangmo *C. oleifera* may be improved according to the international demands for functional nutrients in edible oils.

In this study, we considered four oil quality indices which reflected most of the information on the oil quality of Wangmo *C. oleifera*.*,* and the cumulative variance contribution rate was 76.734% (Table 4). The factor scores and corresponding weights were ranked and summed. The top five germplasms in terms of oil nutritional quality were GH7, GH43, GH28, GH8 and GH31 (Table 5). This ranking provides a guideline for promoting the excellent Wangmo red ball *C. oleifera* plants with high nutritional quality. The final results of this study demonstrated that Wangmo red ball *C. oleifera* seed oil is rich in UFAs, carotenoids, polyphenols, flavonoids, β-sitosterol, squalene and vitamins, and it has dual nutritional and medicinal effects and great potential to be developed into high-quality edible oil.

## Materials and methods

### Experimental site and materials

The experimental site was located in the *Camellia oleifera* germplasm resource nursery in the low-heat valley area of Guizhou University. The climate type was that of the middle and low mountain canyon and the warm and humid climate of the southern subtropical zone. Summer and autumn are controlled by the southwest and southeast monsoons. The summer is rainy and hot, and continuous drought occurs in winter and spring.

The materials used in this study were 20 wild Wangmo *C. oleifera* trees that were observed by the *C. oleifera* team of Guizhou University for five consecutive years (2016–2021). The trees were healthy and free of pests and disease (or with only a few pests) and had high pollination and seed setting rates, stable yield and stable performance. The fruit was collected in late November 2020 in the mature stage, and slight cracking of the peel was a sign considered indicative maturity. Fresh fruits from the selected plants were collected, bagged and labeled for later use.

We adhered to all local, national and international guidelines and legislation for the use of plants in this study.

### Study design

**Figure 3:**
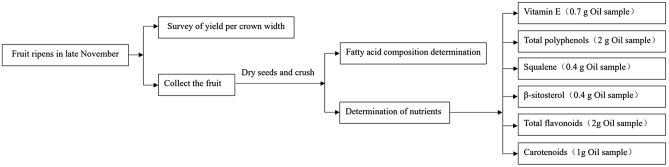
Schematic diagram of the study design.

Figure 3 shows a schematic diagram of the study design, and the fatty acid composition and functional nutrients of Wangmo *C. oleifera* seed oil were assessed comprehensively with the goal of finding a germplasm with excellent comprehensive performance. Briefly, the crown yield of *C. oleifera* was investigated when the fruit was ripe. The fresh fruit was sampled and taken back to the laboratory for drying, and then, the fatty acid and nutritional components were analyzed. Three replicates were performed for each sample.

### Experimental indices

#### Unit crown yield

During the fruit ripening period, the crown width of each *C. oleifera* plant was measured. Fresh fruit was picked and converted to the fresh fruit yield of the whole plant. The yield per unit crown width of each plant was calculated according to the following formula:

$${\text{Yield per unit crown width}}\left( {{\text{kg}}/{\text{m}}^{{\text{2}}} } \right) = \,{\text{Fresh fruit weight of whole}}\;C.\, oleifera\;{\text{plant}}/{\text{crown width of the plant}}.$$

#### Fat content

The sampled fruits were naturally dried. The seeds were collected and then dried in an oven at 40 °C to a constant weight, peeled and then ground with a mortar. Approximately 10 g of the sample was weighed and wrapped in a dried filter paper bag. The bag containing the samples was placed into a Soxhlet extractor (SOX606) for 10-h extraction with petroleum ether. The fat content was calculated as follows:

$${\text{Fat content }}\left( \% \right)\, = \left( {{\text{mass of the extracted fat}}/{\text{that of the sample kernels}}} \right) \times 100\%$$

#### Fatty acid composition analysis

Approximately 100 mg of oil was transferred into a test tube with a ground glass plug, and the oil was methylated using the potassium hydroxide-methanol method. The samples were mixed on a vortex mixer and then oscillated at 40 °C for 30 min. The methyl ester was extracted with 2 ml of n-hexane (analytical grade), and the aqueous phase was discarded. The extract was washed with distilled water and dried with anhydrous sodium sulfate. After filtration, the samples were analyzed with a Shimadzu GC2014 gas chromatograph.

The gas chromatographic conditions were as follows: detector, FID; column type and specification, SP2340; chromatographic column, 60 mx0.25 111/11 × 0.2 um; heating program, 50 °C (initial temperature; retention, 2 min) followed by 170 °C (10 °C/min; retention, 10 min), 180 °C (2 °C/min; retention, 10 min) and 220 °C (4 °C/min; retention, 22 min); injection port temperature (°C), 250; detection temperature (°C), 300. Nitrogen was used as the carrier gas with a shunt ratio of 1:50 and a volume of 1 µL.

The composition of fatty acids was determined based on a comparison with the retention time of various fatty acid standard samples. The relative content of each fatty acid was calculated by the peak area normalization method. The experiment was repeated three times, and average values were obtained.

#### Nutritional substance determination

1. Vitamin E. Oil samples at 0.7 g were accurately weighed, and 10 ml of n-hexane was added for dissolution to a constant volume. The solution was filtered through a 0.22 μM organic filtration membrane for high-performance liquid chromatography (YLSZJ-SB-203).

The liquid chromatographic conditions were as follows: chromatographic column, water amino column; column temperature, 30 °C; mobile phase, n-hexane + isopropanol = 98 + 2; flow velocity, 1.2 mL/min; fluorescence detection wavelength, 295 nm (excitation wavelength) and 333 nm (emission wavelength); amount, 10 μL.

(2) *Total polyphenols* Separate preparations of gallic acid at concentrations of 10, 20, 30, 40 and 50 µg/mL were used. A 2.00 g sample was accurately weighed, dissolved with n-hexane and then passed through the glycol-based column. After purification with 10 mL of n-hexane, the sample was eluted with 10 ml of methanol and then bathed at 45 °C for drying with nitrogen. The sample was redissolved with 50% methanol and then oscillated, chilled and centrifuged. The supernatant was collected. A gallic acid working solution (1 mL), distilled water (1 mL) and the sample to be determined (1 mL) were transferred into a scaled tube. Folin-phenol reagent (0.5 mL), 7.5% sodium carbonate solution (2 mL) and water (6.5 mL) were added. The mixture was subjected to vortex oscillation for 1 min and then bathed at 70 °C for 30 min. The sample was placed on a 10-mm cuvette, and the absorption was read at a wavelength of 750 nm. A standard curve was plotted according to the absorbance of the gallic acid working solution and the concentration of the working solution. Based on the standard curve, the polyphenol concentration was determined, and the content was calculated.
(3) *Squalene and β-sitosterol* An oil sample of 0.4 g was applied to a conical bottle, and 10 mL of KOH-ethanol (2 mol/L) was added. The solution was saponified at 80 °C for 50 min. The unsaponified substances were extracted with n-hexane, washed to neutral, dried and then brought to 2 ml. Gas chromatography was used to calculate the contents of squalene and β-sitosterol with the external standard method.

The chromatographic conditions were as follows: detector, FID; chromatographic column: Agilent HP-5; injection port temperature: 220 °C; heating program, 160 °C for 1 min, which was increased by 15 °C/min to 280 °C for 5 min, followed by an increase by 5 °C/min to 300 °C for 7 min; injection volume: 1 μL.

(4) *Total flavonoids* The 10 mg rutin standard was accurately weighed and brought to 500 mL with 70% ethanol to prepare a 0.2 mg*mL^−1^ standard solution. Standard rutin solutions at 1, 2, 3, 4, 5 and 6 mL, as well as 70% distilled water, were separately prepared. They were transferred into seven 25-ml volumetric flasks. Approximately 1 mL of 5% NaNO_2_ was added. The mixture was oscillated and then allowed to stand for 6 min. Approximately 1 mL of 10% AI(NO3)_3_ was added, and the mixture was oscillated and then allowed to stand for 6 min. Afterward, 10 mL of 4% NaOH was added, and the mixture was brought to volume with distilled water to 25 mL. The mixture was allowed to stand for 15 min. The absorption was read at a wavelength of 510 nm, and the absorption-concentration curve and regression equation were obtained.

The solution to be determined was centrifuged at 6000 r min^−1^ for 10 min. The supernatant (2 mL) was transferred into a 25-mL volumetric flask. As described above, 1 mL of 5% NaNO_2_, 1 mL of 10% AI(NO3)_3_ and 10 mL of 4% NaOH were added sequentially. The mixture was scaled to 25 mL, and then an absorption reading was performed. The formula for the total flavonoid content was as follows:

$$X\left( {g/100g} \right) = \frac{A \times C \times 10}{{m \times v \times 1000}}$$

where *X* is the content of the total flavonoids in the sample, *A* is the content of total flavonoids in the sample extract calculated based on the standard curve, *C* is the size of the sample, *V* is the volume of the solution to be determined, and *m* is the mass of the sample.

(5) *Carotenoids* One gram of oil sample was weighed, and 5 mL of petroleum ether and 5 mL of acetone were added. The absorption of the sample was read at 445 nm, with β-carotene as the standard.

### Statistical analysis

Data were sorted using Excel 2013, and multiple comparisons were performed using SPSS 26.0. The t test was used for comparisons between groups. A difference of *P* < 0.05 was considered statistically significant. After data standardization, principal component analysis (PCA) was performed using SPSS 26.0. The number of principal component factors was determined based on the principle that the eigenvalue of the factor must be greater than 1.00 and the cumulative variance contribution rate must be greater than 80%. After the principal components were extracted, the comprehensive evaluation was carried out, and the comprehensive score *F* was obtained by summing the scores of each principal component with the weight of the variance contribution rate of the corresponding eigenvalue. Origin2018 and ArcGIS were used for plotting.

## Supplementary Information

Supplementary Information 1.

Supplementary Information 2.

## Data Availability

The data used for the analysis in this study are presented in the article and supplementary File 1.
