# Supplementary material for: Fatty acids and nutritional components of the seed oil from Wangmo red ball Camellia oleifera grown in the low-heat valley of Guizhou, China
Source: Sci Rep. 2022 Oct 3;12:16554. doi: 10.1038/s41598-022-20576-y (PMC9530227; doi:10.1038/s41598-022-20576-y)
Supplement: Supplementary file 2 — Supplementary Information 2. [file 41598_2022_20576_MOESM2_ESM.docx]

Supplementary File 1. Raw data of the yield per unit crown width, fat content, fatty acid composition and nutrient composition of the selected plants.
